# The Relation between Fœtus and Pelvis

**Published:** 1902-03-29

**Authors:** 


### THE RELATION BETWEEN FŒTUS AND PELVIS.

From the foregoing remarks we are driven to regard an early diagnosis of abnormalities as the keynote of the midwifery of the future. Dr. Herman points out that with a pelvis of average size, a child of average size, and a normal presentation, there can be no mechanical hindrance to natural delivery. In such a case therefore the whole business of normal labour is the stretching open of the soft parts, and if the genital passage is healthy it will always in time open up. Weak pains will make the labour long but they bring with them no sort of risk. When the pelvis is normal, the child normal, and the genital canal healthy, there is not, and never can be, any necessity for interfering. The very first thing then which we have to do is to discover whether these normal and therefore safe conditions exist, and for this purpose an examination ought to be made at such a time as to give opportunity for preventing the evil consequences of such abnormalities as may be discovered. Medical men, Dr. Herman says, should instruct their patients to come up for examination before the end of the eighth month of pregnancy, and in first pregnancies it would be well to do so at the end of the seventh month. The object of this examination is to ascertain four things: the size of the pelvis; the size of the child; the relative size of the child and the pelvis; and the position of the child. External measurements should be taken, but they are unimportant unless they are much below the average. In regard to internal measurements attempts to reach the sacral promontory will be extremely disagreeable to the patient, and it is therefore better to content oneself with finding out that one cannot reach it without causing pain, and concluding therefore that there is no great pelvic contraction. As to measurement of the fœtus, we cannot do this directly; nor does the size of the abdomen depend upon that of the contained fœtus alone; thus it is impossible to always infer the size of the child from the measurement of the mother's abdomen. Yet there is a mean in these measurments, and it needs no very acute perception to notice that a woman is broader and bigger and fatter than most women. "Women who are bearing children are not generally very fat. The average greatest girth of a woman of medium size at the full term of pregnancy is under 36 inches. . . . The average measurement in such a patient from the symphysis pubis over the convexity of the uterus to the highest point of the fundus is 13 inches. If these measurements are not exceeded the child is not of more than average size, and if we know the pelvis to be of average normal size we may predict a safe and easy delivery so far as mechanical conditions are concerned." The important thing to ascertain is, however, the relative size of the pelvis and the fœtal head, and for doing this Dr. Herman gives the following directions. "The necessary preliminary to ascertaining the relative size of the head and the pelvis is to see that the head is over or in the pelvic brim, to find out the position of the child, and, if the head is not presenting, to turn the child by external palpation so that the head may present. . . . Having ascertained that the head is presenting, or made it present, then see that the child's back is in front, and if it is not, turn the child round (by pressing its shoulders in opposite directions) until its back is in front. Then put the patient in a reclining position, half-way between sitting and lying. In this position the long axis of the uterus will be vertical, and nearly at right angles to the axis of the brim. If, as ought to be the case at eight months' pregnancy, the head is small in relation to the pelvis, it will, by its own weight, sink into the brim. If it does not, then apply the hands, one on each side, with the finger-tips opposite the neck, and then, with the points of the fingers pressing on each end of the fœtal head, press it down into the brim. If the condition of things is normal you can easily do this." All of this is admirable. The next thing to discover is how to persuade the ordinary average woman to take such an intelligent interest in her condition as to submit to and pay for this kind of examination.

